# Cases in Medicine, Interspersed with Strictures, Occasioned by Local Incidents

**Published:** 1782

**Authors:** 


IX. *Cases in Medicine, interspersed with strictures, occasioned by local incidents. By* William Stevenson, *M. D.* The second edition, with corrections and additions. 8vo. *Newark*, 1781. 224 pages.

THE local incidents contained in this work relate to some disputes between our author and the apothecaries at Newark. Dr. Stevenson is of opinion that *Cantharides*, *Tartar Emetic. Mercurius Dulcis*, *Aloes*, *Sena*, *Jalap*, *Salts*, and *Opium*, compose all the efficacy of the apothecary's shop. The rest he considers as " inferior duplicates of these, or fallacious unknown alteratives." " With these—says he—without " scruple, I class the Peruvian bark, that idol " nostrum of the faculty and systematic deceiver " of the world. I have tried it repeatedly and " repeatedly; but with the academical kiss of a " Judas, it has always deceived me. Oak bark " is as good in every medical intention. They " are both but simple bitters, and only do good " as such." In another part of the work we are told, that one physician and one druggist, disinterested in principle and simple in prescription, are sufficient for any district of twenty miles circumference, not including a very large town; town; that the true knowledge of diſorders is compriſable in a ſcore of octavo pages, which now makes huge volumes; and that the remedies for them are reducible to the above-mentioned eight articles.

In the third of the nine caſes related in the work, and which are publiſhed in vindication of his mode of practice, Dr. Stevenſon takes occaſion to declaim with great warmth, and upon the whole with good reaſon, againſt the practice of ſalivation, which he deſcribes as laying a ſure foundation for numberleſs chronical complaints, and ſlowly ſapping the conſtitution. During its violence, he obſerves, it ſtops all the natural ſecretions, patients under ſalivation being almoſt always coſtive, making but little water, and having dry ſkins, till the laſt colliquative ſtools and ſweatings come on, and with which death is continuous.

In the meaſles, Dr. Stevenſon tells us, he has long been accuſtomed to keep a bliſter diſcharging from their very firſt appearance, to preſerve the bowels open all the time, and to allow a dilute proportion of ſpirits and water for the patient's common drink. He aſſures us that by this method the diſeaſe is ſubduable in a few few days, and that all the uſual bad conſequences are entirely prevented.

